# Fundamental Factors Determining the Nature of Parasite Aggregation in Hosts

**DOI:** 10.1371/journal.pone.0116893

**Published:** 2015-02-17

**Authors:** Sébastien Gourbière, Serge Morand, David Waxman

**Affiliations:** 1 Université de Perpignan Via Domitia, EA 4218, ‘Institut de Modélisation et d’Analyse en Géo-Environnements et Santé’ (IMAGES), Perpignan 66100, France; 2 University of Sussex, School of Life Sciences, Centre for the Study of Evolution, Brighton BN1 9QG, UK; 3 Institut des Sciences de l’Evolution—CNRS, Département Génétique Environnement, CC065, Université Montpellier 2, 34095, Montpellier cedex 05, France; 4 Centre for Computational Systems Biology, Fudan University, Shanghai 200433, PRC; NIAID, UNITED STATES

## Abstract

The distribution of parasites in hosts is typically aggregated: a few hosts harbour many parasites, while the remainder of hosts are virtually parasite free. The origin of this almost universal pattern is central to our understanding of host-parasite interactions; it affects many facets of their ecology and evolution. Despite this, the standard statistical framework used to characterize parasite aggregation does not describe the processes generating such a pattern. In this work, we have developed a mathematical framework for the distribution of parasites in hosts, starting from a simple statistical description in terms of two fundamental processes: the exposure of hosts to parasites and the infection success of parasites. This description allows the level of aggregation of parasites in hosts to be related to the random variation in these two processes and to true host heterogeneity. We show that random variation can generate an aggregated distribution and that the common view, that encounters and success are two equivalent filters, applies to the average parasite burden under neutral assumptions but it does not apply to the variance of the parasite burden, and it is not true when heterogeneity between hosts is incorporated in the model. We find that aggregation decreases linearly with the number of encounters, but it depends non-linearly on parasite success. We also find additional terms in the variance of the parasite burden which contribute to the actual level of aggregation in specific biological systems. We have derived the formal expressions of these contributions, and these provide new opportunities to analyse empirical data and tackle the complexity of the origin of aggregation in various host-parasite associations.

## Introduction

A fundamental aspect of the relationship between parasites and hosts is contained in the distribution of parasites amongst hosts. This distribution has repeatedly been shown to be clustered or aggregated (see [1–3] for reviews) in the sense that typically, a few hosts harbour many parasites, while the remainder of the hosts are virtually parasite free. Exceptions to this pattern are so rare that aggregation is considered a fundamental aspect of the definition of parasitism [4] or has been described as the ‘First Law of Parasitism’ [5].

Aggregation has very significant implications for both hosts and parasites, since it affects their genetics and evolution, and has been recognised to have many consequences for public health and livestock management. Aggregation has been shown to affect parasite ecology by stabilizing host-parasite population dynamics [6, 7] and facilitating interspecific co-infection as a result of increased host susceptibility [8]. Aggregation also influences parasite evolution by, e.g., increasing the level of intra-specific competitive interaction and the rate of within-host adaptive parasite diversification [9]. Accordingly, aggregation of parasites amongst hosts affects the transmission of infectious human diseases [10]. Risk heterogeneity typically leads to an increase in R0 of vector-borne diseases by a factor of 2 – 4, and has even larger impact on the transmission of sexually-transmitted diseases, which implies that the proportion of the population that must be protected for elimination via untargeted control program is usually much higher than expected [11]. A quantitative understanding of the mechanisms which lead to the observed levels of aggregation is thus essential for our knowledge of parasite ecology and evolution. One of the most fundamental issues in the field is to what extent differences in parasite loads reflect differences in the exposure of hosts to the infective stages of a parasite, or differences in the success of a parasite in infecting its hosts (see [12], p198).

The distribution of individual parasites amongst hosts is commonly described by a negative binomial distribution since this distribution is a flexible discrete distribution with two parameters [13] whose form naturally accommodates aggregation. However, the negative binomial distribution only provides a phenomenological description of aggregation. In particular, its parameters are not explicitly linked to any representation of the processes underlying exposure of hosts to parasites and the success of the parasites in infecting the hosts. In the present work we introduce a framework, with mechanistic underpinnings, that allows a formal link between the pattern of parasites aggregation amongst hosts, and the processes that lead to this feature.

We proceed by classifying the various processes that are potentially involved in producing the distribution of parasites amongst hosts into two types: (i) the number of encounters between a host and a parasite or a source of parasites, and (ii) the number of parasites that are ultimately carried by a host, which result from a single encounter with a parasite or a source of parasites. These two processes, henceforth referred to as ‘encounters’ and ‘success’ according to Combes conceptual framework (see [12], p. 199), are described by two distributions which combine to produce the overall distribution of the number of parasites in hosts. Because this framework is established with no assumption about the overall distribution of the number of parasites amongst hosts, it gives the ability to test and explore, in a highly flexible way, the relative importance of encounters and success in generating the parasite distribution.

A common assumption in the field is that heterogeneity in hosts generates an aggregated distribution of parasites amongst hosts. Such heterogeneity can be induced by different social or sexual host behaviours that modulate the rate of encounters between susceptible and infected hosts/vectors, or that alter parasite success of infection because of, e.g., allo-grooming (see [14] and [15] for reviews). Variation in physiological status, such as variation in host condition due to nutritional stress, can lead to variations in infections [16, 17]. Finally, habitat difference can generate different risk of contact with the parasite. Typically, inhabitants leaving in the outer part of villages can be more exposed to zoonoses that are transmitted by vectors dispersing from sylvatic areas [18–20]. Thus, without denying the existence of processes that generate heterogeneity in encounters and success, we first question the need for these implicit assumptions and ask: can an aggregated parasite distribution arise without any intrinsic heterogeneity, and simply follow from random variation associated with encounters and success? Given this question we can go further and ask: does randomness in encounters have the same consequences for the parasite distribution as randomness in success? We shall begin the analysis under the very simple assumptions that all hosts are equivalent, and that all parasites are equivalent. This constitutes a neutral model [21] where the parasite distribution in hosts results from random variation in both encounters and success. We then go a step further, and introduce intrinsic differences between hosts, to work out the implications of actual host heterogeneity, which then combines with random variation in encounters and success.

## Results

### Modelling of the parasite distribution

#### Neutral model

The key quantity in this problem is the number of parasites in (or associated with) a host, which we denote by *N*. Since the number of parasites varies from host to host, *N* is a random variable and our model describes the statistics of *N*. Assuming there is no vertical transmission of parasites, the essence of the model is that a host is born parasite free and then has a random number of encounters with a parasite/source of parasites. An encounter is characterised by its success, as measured by the number of parasites that are *ultimately* carried by the host, due to the encounter.

We denote the number of encounters of a host with a parasite/source of parasites by ℰ, and the success of the *j*’th encounter by *S_j_* where *j* = 1, 2, …, ℰ. The number of parasites in a host, *N*, is then given by a sum of successes of the different encounters of the host with a parasite/source of parasites:

$$N=\sum_{j=1}^{ℰ}S_{j}.$$ (1)

We take the *S*_*j*_ and ℰ to be independent random variables whose possible values are 0, 1, 2, … and the value of the sum is taken to zero if ℰ equals zero. The *S*_*j*_ are all taken to have the same probability distribution, and hence have identical expected values and identical variances, which we write as *E*[*S*] and Var(*S*), respectively.

Equation (1) constitutes a fairly general viewpoint for the infection of hosts by a single parasite species. While Eq. (1) looks simple, this appearance is deceptive; *N* is a quantity which is composed of a random number (ℰ) of random variables (the *S*_*j*_) and hence constitutes a *compound random variable* [22], which requires two probability distributions to characterise it. A number of different biological scenarios may be considered for Eq. (1). The variable, ℰ, can represent the number of independent encounters of a given host with individual parasites. Alternatively, ℰ can represent the number of encounters of a host with sources of parasites such as: infected sites (for air, soil or water-borne diseases), infected vectors (for vector-borne diseases) or infected hosts for directly transmitted parasites. Similarly, the variable *S*_*j*_ could represent the viability of a single parasite, and hence would only take two values, namely 0 or 1, corresponding to death or survival of the parasite. However, the variable *S*_*j*_ could also account for multiple infections/contacts and/or within host parasite viability and reproduction, in which case *S*_*j*_ is capable of taking values larger than 1, and the discrete distribution it follows would reflect this feature.

It is worth mentioning that the causes of the random variability in success can be associated with the parasite or with the host. Such variability can be due to a finite random sampling of parasite diversity in each host, just as it can reflect random variability in host resistance or condition. But, whatever the definition of ℰ and *S*_*j*_ and their causes, the key assumption of this neutral model is that the levels of random variability in encounters and success are the same for all hosts. In other words, encounters and success are realizations of random variables whose distribution is common to all individuals, and hosts are thus ‘statistically equivalent’. Accordingly, there is no intrinsic difference between hosts and these variations can be seen as a form of demographic stochasticity, whose effects on parasite distribution can be evaluated from the neutral model.

#### Heterogeneous model

The above model can now be expanded to incorporate host heterogeneity, by allowing different types of hosts, labelled *t* = 1, 2 … *h*. Consider a randomly captured host of type *t*. The number of parasites in the host is then related to the following: (i) the number of encounters, specific to a host of type *t*, that the host has with a parasite/source of parasites, and which we write as ℰ_*t*_, and (ii) the success that is associated with each encounter of a host of type *t*, which we write as $S_{j}^{\left(t\right)}$. The number of parasites in a randomly captured host can be written as

$$N=\sum_{t=1}^{h}H_{t}\sum_{j=1}^{{ℰ}_{t}}S_{j}^{\left(t\right)}.$$ (2)

In this equation the *H*_*t*_ indicate the type of host captured. Only one of the *H*_*t*_’s takes the value of unity, while the remainder are zero, and the probability with which *H*_*t*_ = 1 is *p*_*t*_. Thus explicitly, (*H*_1_, *H*_2_, …, *H*_*h*_) constitutes a multinomial random variable with parameters 1 and (*p*_1_, *p*_2_, …, *p*_*h*_).

As in the neutral model, there are various alternative biological scenarios that can lead to heterogeneity in the rate at which different hosts encounter parasites. The rate of encounters can vary with the level of host foraging activities [23, 24], the characteristics of the host habitat [25–27], the spatial and temporal co-distributions of hosts and vectors [28–31]. Similarly, the rate of parasite success in the host can depend on the individual level of immunity [32–34] or physiological status [35–38].

### Results for the specific models

We shall establish general results for some summary statistics of the distribution of parasites in hosts, that emerges from the neutral and heterogeneous models introduced above.

#### Neutral model

All results given below, for the neutral model, are established in Methods A.

We begin with Eq. (1), which relates the number of parasites of an individual host, *N*, to the number of encounters that they have with a parasite/source of parasites, ℰ, and the ultimate success of these parasites on different encounters, i.e., the *S*_*j*_. A direct consequence of Eq. (1) combined with the assumptions made above is that the expected value of the number of parasites of a host, *E*[*N*], is related to the expected number of encounters, *E*[ℰ], and the expected level of success, *E*[*S*], as

E[N]=E[ℰ]E[S]. (3)

Additionally, the relationship between the variance of *N* and the various statistics of ℰ and *S*_*j*_ can be shown to be

$$\text{Var}(N)=\text{Var}(ℰ){\left(E[S]\right)}^{2}+\text{Var}(S)E[ℰ].$$ (4)

Equations (3) and (4) are standard results for compound random variables (see e.g., [22]), and can be found in Methods A, as part of the analysis.

It is important to point out that Eqs. (3) and (4) apply for any distributions of ℰ and *S*_*j*_, and hence for any distribution of *N* that results from Eq. (1). In particular, Eqs. (3) and (4) apply whether or not *N* has a negative binomial distribution. This is important since, using our framework, it can be shown that conditions need to be satisfied, by the distributions of encounters and success, in order to obtain a negative binomial distribution (see Methods A). These conditions appear to be rather specific for the distribution of successes, as we show in the second example in Methods A.

As expected, the mean number of contacts and successes combine in a symmetric way to produce the mean number of parasites in a host [12]. However, the asymmetric way that the summary statistics of encounters and success enter the result for the variance in the number of parasites in a host, Eq. (4), means that these two processes cannot be regarded as having equivalent effects on the variance.

We note that the variance of *N* depends on the variance of the number of contacts and the variance of success, however, these variances are weighted differently in Eq. (4), namely by the squared mean of success and the mean of contact, respectively. Such an asymmetry of the weightings allows the distribution of parasites in hosts to take a variety of forms. We thus used a measure of aggregation that is applicable to a general distribution, namely the variance-to-mean-ratio of the number of parasites in hosts. Values of the variance-to-mean-ratio that are greater than 1 are associated with aggregation [39]. Using Eqs. (3) and (4) this ratio can be expressed as the sum of two terms, involving the variance-to-mean-ratio in encounters and success:

$$\frac{\text{Var}(N)}{E[N]}=\frac{\text{Var}(ℰ)}{E[ℰ]}E[S]+\frac{\text{Var}(S)}{E[S]}.$$ (5)

**Figure 1:**
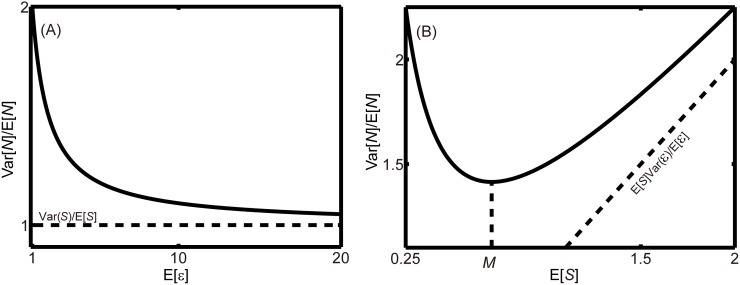
This figure illustrates how aggregation varies with either host-parasite encounters or parasite success (in infecting hosts). In Panel A we have adopted the values Var(*S*)/*E*[*S*] = 1 and Var(ℰ)*E*[*S*] = 1. We show how parasite aggregation varies with the mean number of encounters. The level of aggregation decreases with the number of encounters, and asymptotically approaches a value that depends only on the variance-to-mean ratio of parasite success, i.e., Var(*S*)/*E*[*S*]. In Panel B we have adopted the values Var(*ℰ*)/*E*[*ℰ*] = 1 and Var(*S*) = 0.5. We show how parasite aggregation varies with the mean parasite success in infecting hosts. The level of aggregation initially decreases with the average success of parasites in infecting their hosts until a minimum is reached at a value of *E*[*S*] of $M=\sqrt{\text{Var}(S)E[ℰ]/\text{Var}(ℰ)}$, as indicated on the abscissa. The level of aggregation then starts to increase, with an asymptotically achieved slope that is directly proportional to the variance-to-mean-ratio of encounters, i.e., Var(ℰ)/*E*[ℰ].

The two contributions in this equation, of the variance-to-mean-ratios of encounters and success, are weighted differently; the average success multiplies the contribution of the variance-to-mean-ratio in encounters. The simplicity of this quantitative outcome of our neutral model leads to two general insights into the emergence of aggregation from random variation associated with encounters and success. First, aggregation decreases with the mean number of contacts, *E*[ℰ], until the level of aggregation reaches an asymptotic level of Var(*S*)/*E*[*S*], and only reflects randomness in success (Fig. 1A). Second, the mean level of success, *E*[*S*], can have a more complex effect on aggregation than the mean number of contacts because of its presence in two places in Eq. (5) (Fig. 1B).

Any increase in *E*[*S*] (at a fixed value of Var(*S*)) decreases the variance-to-mean-ratio of success, and thus lowers the level of aggregation. However, increasing *E*[*S*] simultaneously increases the contribution of randomness in encounters by magnifying the differences between hosts that had low and high levels of contacts with parasites. The balance between these two antagonistic effects leads to an intermediate level of average success of $E[S]=\sqrt{\text{Var}(S)E[ℰ]/\text{Var}(ℰ)}$ that corresponds to a lowest level of aggregation of $2\sqrt{\text{Var}(S)\text{Var}(ℰ)/E[ℰ]}$, while at larger *E*[*S*] the level of aggregation asymptotically changes as *E*[*S*]Var(ℰ)/*E*[ℰ].

Although we aim at providing a mechanistic alternative to the negative binomial distribution to describe parasite distributions, it is worth mentioning that the standard measure of aggregation, the parameter *k* of the negative binomial distribution distribution, can be expressed in terms of the summary statistics of ℰ and *S*_*j*_ (see Methods A). This could be used when the specific conditions for the distribution to be a negative binomial distribution are fulfilled (see Methods A).

#### Heterogeneous model

All results given below, for the heterogeneous model, are established in Methods B.

The above results can be expanded to multiple types of hosts by using Eq. (2) that relates the number of parasites of an individual host, *N*, to random variables associated with encounters and success, for different host types. The mean and variance of the number of encounters are then conditional on the host type, and we write these as *E*[ℰ∣*t*] and Var(ℰ∣*t*), respectively. Similarly, we denote the mean and variance of the success of a parasite to infect hosts of type *t* by *E*[*S*∣*t*] and Var(*S*∣*t*), respectively. We find that the expected number of parasites in a host is a weighted average over contributions from different possible host types:

$$E[N]=\sum_{t=1}^{h}p_{t}E[ℰ|t]E[S|t]$$ (6)

(see Methods B for details) where *p*_*t*_ denotes the frequency of host type *t*. Thus, the mean number of parasites in a host depends on the mean number of encounters and success in each host type and on the way these are correlated across host types. Intuitively, we expect the total number of parasites to be larger when the hosts with the highest rate of encounters with parasites are the more susceptible ones. This appears more obviously in an alternative expression for *E*[*N*] that is completely equivalent to Eq. (6), namely

E[N]=μm+Cov(ℰ,S). (7)

Here $\mu =\sum_{t=1}^{h}p_{t}E[ℰ|t]$ and $m=\sum_{t=1}^{h}p_{t}E[S|t]$ represent the means of encounters and successes, across different host types, while $\text{Cov}(ℰ,S)=\sum_{t=1}^{h}p_{t}\left(E[ℰ|t]-\mu \right)\left(E[S|t]-m\right)$ measures how encounter and success are correlated with each other across different host types. Equation (7) clearly shows that the mean number of parasites is linearly related to the covariance between encounters and success.

A negative correlation between encounters and infection success can emerge as a result of processes related to host group size. The rate of encounter with directly transmitted parasites is positively correlated with the size of the group in many host species [40]. But at the same time, infection success of ectoparasites may decrease with the number of individuals through enhanced allo-grooming behaviour [31, 41]. Alternatively, a positive correlation can result from variation associated with home range. A small home range size in mammals is usually associated with increased host densities that favours parasite encounters and simultaneously affects host condition, which has a positive effect on infection success [16].

The variance of the distribution of parasites in hosts, Var(*N*), that incorporates random individual variation and actual heterogeneity in contacts and success can also be derived (see Methods B) and is given by

$$\text{Var}(N)\;=\overset{h}{\underset{t=1}{\sum }}\;p_{t}\left[\text{Var}(ℰ|t){\left(E[S|t]\right)}^{2}+E[ℰ\left|t]\text{Var}(S\right|t)\right]+\overset{h}{\underset{t=1}{\sum }}\;p_{t}{\left(E[ℰ\left|t]E[S\right|t]-\overset{h}{\underset{u=1}{\sum }}\;p_{u}E[ℰ\left|u]E[S\right|u]\right)}^{2}.$$ (8)

This result indicates that there are contributions to Var(*N*) from individual variation that is within host types (first sum), and also from effects of heterogeneity between host types (second sum).

The expression in Eq. (8) can be approximated, to provide a more explicit relationship between the variance in parasite numbers in hosts and various correlations involving encounters and success. We achieve this by determining the leading corrections to the results of the homogeneous model (see Methods B for details). This leads to an equation for the variance in the number of parasites in a host of the form

$$\text{Var}(N)\approx \sigma^{2}m^{2}+\mu \nu^{2}+\text{quadratic}\;\text{correlation}\;\text{terms}$$ (9)

where $\sigma^{2}=\sum_{t=1}^{h}p_{t}\text{Var}\;\left(ℰ|t\right)$ and $\nu^{2}=\sum_{t=1}^{h}p_{t}\text{Var}\;\left(S|t\right)$ stand for means, across all host types, of the variances of encounter and success. The leading two terms in Eq. (9) are equivalent to the terms in the homogeneous model, Eq. (4), so that conclusions on the asymmetric contributions of the levels of contact and success remain, although they now apply to averages across host types. Obviously, the quadratic correlation terms can potentially make the whole expression of Eq. (9) substantially more complicated, as one should expect from the level of complexity incorporated into the heterogeneous model. However, these additional terms can be fully identified from Eqs. (B3) and (B9) of Methods B, and they are related to the variances and covariances of the basic summary statistics (mean and variance) describing the distribution of encounters and success conditional on host types (see Eq. (B10) in Methods B).

The variance-to-mean-ratio of the number of parasites amongst hosts can, using Eqs. (7) and (9), be approximated as

$$\frac{\text{Var}(N)}{E[N]}=\frac{\sigma^{2}m^{2}+\mu \nu^{2}}{\mu m}+\text{quadratic}\;\text{correlation}\;\text{terms}$$ (10)

where, as above, the quadratic correlation terms take into account leading effects of between-type correlations. Thus again, the leading terms in Eq. (10) on the right hand side are equivalent to the results of the homogeneous model (Eq. (5)), although they now apply to means across host types. Accordingly, the highest levels of aggregation are expected when strong levels of random individual variation in encounters (*σ* ^2^) are associated with high average rates of success (*m*) as the latter magnifies the former. Similarly, aggregation increases when there is large random individual variation in success (*ν* ^2^) are associated with high average rates of encounters (*μ*). It is not obvious how to identify host-parasite systems that could serve as examples of such co-variations since random individual variation in encounters and success are typically not measured in the field. However, there are a few published experimental results that suggest that rodents exposed to water-borne, air-borne or even soil-transmitted parasites could be good candidates for measurement of the level of such demographic stochasticity (see discussion). As expected, parasites will also be strongly aggregated when hosts with a high average rate of encounters are those on which parasites are successful, since this would lead to high mean parasite load and a lower variance to the mean ratio.

As explained above, such a positive correlation could be associated with a small host home range, while a negative correlation is expected in host groups of large size.

The quadratic correlation terms of Eq. (10) are directly related to those appearing in Eq. (B12) of Methods B, and thus also correspond to variances and covariances of basic summary statistics of the distributions of encounters and success. These expressions make up an explicit link between the patterns of aggregation and basic statistics on the level of host random individual variation and actual heterogeneity in the processes of encounters and success. Although no general results can be derived from these quadratic correlations because of their remaining complexity, Eq. (9) and (10) provide the theoretical background necessary to gain a better knowledge of the parasite distribution, if combined with a good empirical knowledge of the undoubtedly system-specific correlations between encounters and success.

## Discussion

Aggregation of parasites amongst hosts is one of the rare phenomena in biology that has been described as a ‘law’ [1, 5]. The negative binomial distribution has been of central importance to establish evidence for aggregation in data [5, 42], as well as to provide theoretical predictions on ecological and evolutionary consequences of parasite distributions among hosts [6, 9, 43]. However, a good fit to a negative binomial distribution does not provide any information about the causes of aggregation since a number of different biological phenomena have been proposed to generate this flexible distribution [44], as well as different combinations of statistical laws [13]. A good fit cannot then be interpreted as support of a unique hypothesis about any underlying mechanisms. In the present work, we have introduced and developed simple mechanistic models of parasite aggregation, which are taken to originate from distributions corresponding to the two main types of factors thought to generate aggregation: (i) the encounters between host and parasites, and (ii) the success of parasites once in contact with the host [12]. This provides a biologically intuitive mathematical framework to quantitatively investigate the importance of random individual variation and actual heterogeneities in encounters and success on parasite aggregation. Here we discuss the main outcomes of the model and the way they could be tested empirically.

A first outcome of the present work is that random individual variation can potentially produce an aggregated distribution of parasites amongst hosts. Such prediction is consistent with previous empirical and theoretical finding, where causes other than intrinsic heterogeneity amongst hosts generate aggregated distributions of parasites. The rate of mice infection by blacklegged ticks depends substantially on ‘bad luck’, i.e. inhabiting a home range with high vector density [28]. Aggregated distributions have also been shown to emerge when parasites are homogeneously distributed in the environment, provided that the probability of infection is related to the distance between the host and the source of parasites [45]. Additionally, clumped infections, i.e. infections of several larvae at the same time, have a strong impact on the level of aggregation [46]. We finally note that similar aggregated distributions of parasites [47] and parasitoids [48] have been generated using compound distributions representing other more specific demographic processes. One cannot thus a priori rule out the null hypothesis that random variation is partly responsible for the observed levels of aggregation (see [1–3] for reviews), and the causal relationships between sources of true heterogeneity amongst hosts and the observed distribution of parasites should thus be better quantified by taken into account random individual variation in encounters and success. An obvious need is to evaluate the level of ‘neutral aggregation’ that can be explained in absence of actual host heterogeneity. Our neutral model allows for such evaluation provided that it can be parameterized from independent experimental assessments of the levels of random individual variation in encounters and success. Dose-infectivity curves derived from artificial infection experiments could allow the measurement of variability in infection success in the same way as they have been used to assess the distribution of host susceptibility [49–51]. Some of these authors [51] then used a modelling framework that assumed a constant number of encounters with parasites, and a flexible statistical distribution to describe host variation in the rate of parasite acquisition. Fitting this relationship to dose-infectivity profile they obtained the maximum likelihood estimates of the parameters of the host susceptibility distribution. Setting up similar experiments with all host individuals originating from a single type, e.g. isogenic mice, the outcome would provide a distribution that gives a measure of random individual variation in parasite success. To estimate random individual variation in encounters may be more challenging, but could be investigated whenever hosts can be kept in parasite free environments and the rate of exposure controlled experimentally. For instance, individually marked rainbow trout were introduced at regular time intervals into cages so that the rate of exposure to trematode parasites, under natural conditions, could be controlled [52]. Similar experimental designs are conceivable for rodents that can be breed and exposed at controlled rate to water-borne (such as the fluke Schistosoma mansoni, [53]), to food borne (such as the acanthocephalan Moniliformis monoliformis through the consumption of infected prey, [54]) or even soil-transmitted parasites (such as Nippostrongylus brasiliensis, [55]). Interestingly, our results also suggest that to assess individual variation in one of these two components (encounter or success), the experimental design should be set up with a high average level of the other component. We note that when the average number of encounters is large, the level of parasite aggregation converges toward the level of random variation in success (Var(*N*)/*E*[*N*] ∼ Var(*S*)/*E*[*S*]). Similarly, when the level of parasite success is large, the level of parasite aggregation is linearly related to the level of random variation in contacts (Var(*N*)/*E*[*N*] ∼ Var(ℰ)*E*[*S*]/*E*[ℰ]). Under these conditions, the desired quantities could thus be more easily assessed.

A second significant outcome of our model is in providing simple predictions in the relative effect of randomness in encounters and success on the parasite distribution. Although the mean values of encounter and success combine multiplicatively to give the mean number of parasites in hosts, as expected when we take the view that the ‘filter’ of encounter and the ‘filter’ of compatibility are equivalent ‘apertures’ that limit the acquisition of parasites by hosts [12], the latter representation is misleading when trying to understand the variability of infection and thus aggregation. As a consequence of the natural sequential order in which encounter and success contribute to determine the parasite load of a host, the two ‘filters’ are not equivalent in their effects on the variance-to-mean-ratio in the number of parasites in hosts. While aggregation decreases with the average number of host-parasite encounters and converges towards Var(*S*)/*E*[*S*], it varies in a non-linear way with the average parasite success in hosts. This results in an intermediate level of success, equal to $\sqrt{E[ℰ]\text{Var}(S)/\text{Var}(ℰ)}$, leading to the lowest level of aggregation. Such a prediction could be tested in the above experimental settings by controlling the level of average success in hosts through the use of various strains of parasites [56], manipulation of the physiological status of the host [57] or change in its level of immunity [58].

Importantly, the asymmetry between the effect of encounters and success is also apparent in the contributions of actual host heterogeneity. Although some general insights can be drawn from our results, effects are then more complex and hard to anticipate without a specific system at hand. Investigation of those effects should be started on very simple systems with a known (and simple) source of heterogeneity, like resistant, sensitive or even tolerant host genotypes [59]. In such context, and in an experimental set up similar to those discussed above, one would be able to measure the host type specific average rates of contact and success as well as the level of random variation in success and contact for each type. This could provide estimates of terms appearing in Eqs. (9) and (10) would give the opportunity to handle standard sensitivity or elasticity analysis to clarify the contributions of random individual variation and actual differences between hosts in generating aggregation in specific systems.

To conclude, the mechanistic framework that we have developed in this paper formally links the pattern of parasite aggregation to random variation and heterogeneities in the processes of host-parasite encounters and the success of infection. This simple but flexible framework should improve our ability to gain a better understanding of the origins and implications of aggregation in parasite ecology, evolution and the control of infectious diseases.

## Methods

### A. Derivation of results for the neutral model

In this subsection we give a derivation of results for the neutral model. We first determine general results (Eqs. (3) and (4) of the main text) before showing particular results related to the use of the negative binomial distribution. We note that there may sometimes need to be constraints on the distributions of ℰ and *S*_*j*_.

#### General results

We begin with a discrete random variable *N*, which takes the values 0, 1, 2, …. The *probability generating function* for *N* is defined by $G_{N}(\lambda )=E[\lambda^{N}]=\sum_{n=0}^{\infty }\text{Prob}(N=n)\lambda^{n}$ where *E*[…] denotes an expected (or average value) and *λ* is a real variable. All information about the distribution of *N* is contained in *G*_*N*_(*λ*) [22]. Henceforth we shall refer to probability generating functions simply as *generating functions*.

We take *N* to represent the number of parasites in a randomly picked host, as given by Eq. (1) of the main text, namely $N=\sum_{j=1}^{ℰ}S_{j}$ where ℰ is the number of exposures of a host to a parasite/source of parasites and *S*_*j*_ is the ultimate success of a parasite on the *j*’th exposure. The value of *N* is taken to be 0 if ℰ = 0. We assume ℰ and the *S*_*j*_ are all independent random variables that can take the values 0, 1, 2, …. We take all *S*_*j*_ to have identical distributions, with expected value *E*[*S*] and variance Var(*S*). The generating function of *N*, that follows from Eq. (1), is [22]

$$G_{N}(\lambda )=G_{ℰ}\left(G_{S}(\lambda )\right).$$ (A1)

Differentiating Eq. (A1) once or twice with respect to *λ* and then setting *λ* = 1 leads to results for *E*[*N*] and *E*[*N*(*N* − 1)] that correspond to the following relations between the means and variances of *N*, ℰ and *S*:

$$\begin{array}{cc} E[N] & =E[E]E[S]\\ \text{Var}(N) & =E[E]\text{Var}(S)+\text{Var}(E){\left(E[S]\right)}^{2}. \end{array}$$ (A2)

#### Particular results on the negative binomial distribution

To establish some particular but informative results, we take *N* to have a negative binomial distribution. The form of this distribution that we use follows Anderson and May [6]. It has parameters *m* and *k* and is defined by

$$\begin{array}{cc} \text{Prob}(N=n) & \equiv B(n;m,k)\\ & ={\left(\frac{k}{k+m}\right)}^{k}\frac{\Gamma (k+n)}{\Gamma (k)\Gamma (n+1)}{\left(\frac{m}{k+m}\right)}^{n},\;n=0,1,2,\ldots \end{array}$$ (A3)

where Γ(*x*) denotes Euler’s Gamma function. This distribution has a mean of *E*[*N*] = *m* and a variance of Var(*N*) = *m* ^2^/*k* + *m*. The parameter *k* is often used as a measure of aggregation of the distribution and it can be approximated as

$$k=\frac{{\left(E[N]\right)}^{2}}{\text{Var}(N)-E[N]}.$$ (A4)

#### Constraints on the distribution of ℰ and S that lead to a negative binomial distribution

It is convenient to express the generating function of *N* in terms of the parameter

r=m/k (A5)

and then find that

$$G_{N}(\lambda )=E[\lambda^{N}]={\left(1+r-\lambda r\right)}^{-k}.$$ (A6)

When *N* has the distribution of Eq. (A3), the equation of the generating function, Eq. (A1), imposes a relationship between the generating functions *G*_ℰ_(*λ*) and *G*_*S*_(*λ*), namely

$${\left(1+r-\lambda r\right)}^{-k}=G_{ℰ}\left(G_{S}\%(\lambda )\right).$$ (A7)

Equivalently, this equation expresses a relationship between the probability distributions of ℰ and *S*_*j*_. We note that if we specify either *G*_ℰ_(*λ*) or *G*_*S*_(*λ*), we can exploit Eq. (A7) to determine the other generating function. This generating function can then be used to determine the corresponding probability distribution. Note however, that this procedure does not always work: it sometimes gives rise to an invalid probability distribution, because some of the ‘probabilities’ obtained are negative. We give two illustrative examples of this usage of Eq. (A7). In the first example we determine the distribution of ℰ, after assuming the distribution of *S*. In the second example, the distribution of *S* is determined after assuming the distribution of ℰ. In this case we find that a probability distribution for *S* follows only for restricted values of some of the parameters.

**Example 1**

If *S* can only take the values 0 and 1, and these occur with probabilities 1 − *α* and *α* respectively, then *G*_*S*_(*λ*) = 1 − *α* + *αλ*. It follows from Eq. (A7) that (1 + *r* − *λr*)^−*k*^ = *G*_ℰ_(1 − *α* + *αλ*). Solving this equation for *G*_ℰ_(*λ*) yields *G*_ℰ_(*λ*) = (1 + *r*/*α* − *λr*/*α*)^−*k*^ which, on comparison with Eq. (A6), corresponds to ℰ having a negative binomial distribution with parameters *m*/*α* and *k*:

Prob(ℰ=ε)=B(ε;m/α,k),  ε=0,1,2,… (A8)

This example leads to *E*[ℰ] = *m*/*α*, *E*[*S*] = *α*, Var(ℰ) = *m*/*α* + *m* ^2^/(*α* ^2^ *k*) and Var(*S*) = *α*(1 − *α*).

**Example 2**

If ℰ has a Poisson distribution, with Prob(ℰ = *n*) = *β* ^*n*^ *e* ^−*β*^/*n*! for *n* = 0, 1, 2, … then *G*_ℰ_(*λ*) = exp(*βλ* − *β*). Using this result in Eq. (A7) yields (1 + *r* − *λr*)^−*k*^ = exp(*βG*_*S*_(*λ*) − *β*), and this can be directly solved for *G*_*S*_(*λ*) with the result *G*_*S*_(*λ*) = 1 − (*k*/*β*) ln (1 + *r* − *λr*). Expanding *G*_*S*_(*λ*) in powers of *λ* leads to

$$\text{Prob}(S=s)=\{\begin{array}{lll} 1-\frac{k}{\beta }\ln\left(1+m/k\right), & & s=0\\ \frac{k}{\beta }{\left(\frac{m}{m+k}\right)}^{s}\frac{1}{s}, & & s=1,2,3,\ldots \end{array}$$ (A9)

This distribution can be viewed as a mixture of two distributions: a unit probability mass located at *s* = 0 with weight 1 − (*k*/*β*) ln (1 + *m*/*k*), and a logarithmic distribution with overall weight (*k*/*β*) ln (1 + *m*/*k*). The values of the parameters *m*, *k* and *β* cannot take any values, but must be chosen so the probabilities of different values of *S* (in particular *S* = 0) are all non-negative. For example, at fixed values of *k* and *m*, the parameter *β* must have some restrictions placed upon it; in this case we can only consider values of *β* which yield 1 − (*k*/*β*) ln (1 + *m*/*k*) ≥ 0 so that Prob(*S* = 0) ≥ 0. Defining

$$\beta_{\min}=k\ln\left(1+m/k\right)$$ (A10)

we thus require *β* ≥ *β*_min_ for the probabilities of all values of *S* to be non-negative.

This example leads to *E*[ℰ] = *β*, *E*[*S*] = *m*/*β*, Var(ℰ) = *β* and Var(*S*) = (*m*/*β*)(1 + *m*/*k* − *m*/*β*).

### B. Derivation of results for the heterogeneous model

In this subsection we present a derivation of results for the heterogeneous model that was introduced in this work.

In the heterogeneous model, there are *h* different types of hosts. In general, each host type has a different distribution of encounters with a parasite/source of parasites, and the parasite has a different distribution of success on each host type. The number of parasites, *N*, in a randomly picked host is taken to be

$$N=\sum_{t=1}^{h}H_{t}\sum_{k=1}^{{ℰ}_{t}}S_{k}^{\left(t\right)}.$$ (B1)

In this equation $\text{H}\overset{\text{def}}{\equiv }(H_{1},H_{2},...,H_{h})$ constitutes a multinomial random variable with parameters 1 and (*p*_1_, *p*_2_, …, *p*_*h*_). Each *H*_*t*_ can only take the values 0 and 1, and in a realisation of **H** only one of the *H*_*t*_’s equals 1 (corresponding to the particular host type obtained) while the rest are 0. The probability with which *H*_*t*_ = 1 is *p*_*t*_.

To indicate that expected values (and other statistics) are conditional on host type, we shall use notation where *E*[ℰ∣*t*] and Var(ℰ∣*t*) denote the mean and variance of the number of encounters, when conditional on the host type *t*.

Proceeding, we note that with *N* given by Eq. (B1), and *a*_1_, *a*_2_, …, *a*_*h*_ a set of non random numbers, we can determine the generating function of *N*, using the result $E\left[\lambda^{\sum_{t=1}^{h}H_{t}a_{t}}\right]=\sum_{t=1}^{h}p_{t}\lambda^{a_{t}}$. This leads to

$$G_{N}\left(\lambda \right)=E\left[\lambda^{\sum_{t=1}^{h}H_{t}\sum_{k=1}^{E_{t}}S_{k}^{\left(t\right)}}\right]=\sum_{t=1}^{h}p_{t}E\left[\lambda^{\sum_{k=1}^{E_{t}}S_{k}^{\left(t\right)}}\right]=\sum_{t=1}^{h}p_{t}G_{E_{t}}\left(G_{S^{\left(t\right)}}\left(\lambda \right)\right).\;\left(B2\right)$$ (B2)

We shall determine the mean and variance of *N* from this expression, and it is helpful to establish some notation that we shall use. We define

$$\begin{array}{cc} \mu & =\sum_{t=1}^{h}p_{t}E[E|t]\\ m & =\sum_{t=1}^{h}p_{t}E[S|t]\\ \sigma^{2} & =\sum_{t=1}^{h}p_{t}\text{Var}(E|t)\\ \nu^{2} & =\sum_{t=1}^{h}p_{t}\text{Var}(S|t)\\ C_{a,b,c,d} & =\sum_{t=1}^{h}p_{t}{\left(E[E|t]-\mu \right)}^{a}{\left(\text{Var}(E|t)-\sigma^{2}\right)}^{b}{\left(E[S|t]-m\right)}^{c}{\left(\text{Var}(S|t)-\nu^{2}\right)}^{d} \end{array}$$ (B3)

#### Mean of *N*

We determine the mean of *N* by differentiating *G*_*N*_(*λ*) once with respect to *λ* and then setting *λ* = 1. This yields

$$E[N]=\sum_{t=1}^{h}p_{t}E[ℰ|t]E[S|t].$$ (B4)

Equation (B4) can be rewritten in the alternative form

$$E[N]=\mu m+C_{1,0,1,0}$$ (B5)

where *C*_*a*, *b*, *c*, *d*_ is defined in Eq. (B3) and explicitly, *C*_1, 0, 1, 0_ is the covariance

$$C_{1,0,1,0}=\overset{h}{\underset{t=1}{\sum }}p_{t}\left(E[ℰ|t]-\mu \right)\left(E[S|t]-m\right).$$ (B6)

#### Variance of *N*

We first determine the expected value *E*[*N*(*N* − 1)] by differentiating *G*_*N*_(*λ*) (Eq. (B2)) twice with respect to *λ* and then setting *λ* = 1. This yields

$$E\left[N\left(N-1\right)\right]=\overset{h}{\underset{t=1}{\sum }}\;p_{t}E[ℰ\left(ℰ-1\right)|t]{\left(E[S|t]\right)}^{2}+\overset{h}{\underset{t=1}{\sum }}\;p_{t}E[ℰ|t]E[S\left(S-1\right)|t].$$ (B7)

From this result and Eq. (B4) we obtain

$$Var\left(N\right)=\overset{h}{\underset{t=1}{\sum }}\;p_{t}\;Var\left(ℰ|t\right){\left(E[S|t]\right)}^{2}+\overset{h}{\underset{t=1}{\sum }}\;p_{t}E[ℰ|t]Var\left(S|t\right)+\overset{h}{\underset{t=1}{\sum }}\;p_{t}{\left(E[ℰ|t]E[S|t]-\overset{h}{\underset{i=1}{\sum }}\;p_{i}E[ℰ|i]E[S|i]\right)}^{2}.$$ (B8)

Hence having multiple types leads to an extra level of averaging (as indicated by the sums in Eq. (B8)) plus an additional positive term in the variance reflecting an aspect of variation between different host types (the final sum in Eq. (B8).

The expression in Eq. (B8) is somewhat complicated, but we can approximate it, under the assumption that deviations *between* different host types that are higher than quadratic order can be neglected. Using the *delta technique* (see e.g., [60]), we obtain the expression

$$\text{Var}(N)\;\;\approx \sigma^{2}m^{2}+\mu \nu^{2}+\left(\sigma^{2}+\mu^{2}\right)C_{0,0,2,0}+m^{2}C_{2,0,0,0}+2\mu mC_{1,0,1,0}+2mC_{0,1,1,0}+C_{1,0,0,1}$$ (B9)

which contains a leading term that is derived from average over all types, and various quadratic correlation terms that arise from between type variation.

We can write the *C*_*a*, *b*, *c*, *d*_ that appear in Eq. (B9) in a more suggestive form, using *T* to denote a random variable whose value corresponds to the type of host captured. We then have

$$\begin{array}{l} C_{0,0,2,0}=Var\left(E[S|T]\right)\;\\ C_{2,0,0,0}=Var\left(E[ℰ|T]\right)\\ C_{1,0,1,0}=\text{Cov}\;\left(E\left[ℰ\left|T],E[S\right|T\right]\right)\\ C_{0,1,1,0}=\text{Cov}\;\left(Var\left(ℰ|T\right),E[S|T]\right)\\ C_{1,0,0,1}=\text{Cov}\;\left(E[S|T],Var(E[S|T])\right). \end{array}$$ (B10)

Using the above forms for the *C*_*a*, *b*, *c*, *d*_ we can write Eqs. (B5) and (B9) as

E[N]=μm+Cov(E[ℰ|T],E[S|T]) (B11)

$$Var\left(N\right)\approx \sigma^{2}m^{2}+\mu \nu^{2}+\left(\sigma^{2}+\mu^{2}\right)Var\left(E[S|T]\right)+m^{2}Var\left(E[ℰ|T]\right)+2\mu m\text{Cov}\;\left(E\left[ℰ\left|T],E[S\right|T\right]\right)+2m\text{Cov}\;\left(Var\;\left(ℰT\right),E[S|T]\right)+\text{Cov}\;\left(E[S|T],Var(E[S|T])\right).$$ (B12)
